# Effect of Protein, Carbohydrate, and Oil on Phytochemical Bioaccessibility and Bioactivities of the *Ginkgo biloba* L. Leaf Formulations After In Vitro Digestion

**DOI:** 10.3390/molecules29225300

**Published:** 2024-11-09

**Authors:** Gordana Rusak, Valerija Vujčić Bok, Ivana Šola, Ema Nikša, Željan Maleš

**Affiliations:** 1 Department of Biology, Faculty of Science, University of Zagreb, Horvatovac 102a, 10000 Zagreb, Croatia; gordana.rusak@biol.pmf.hr (G.R.); ivana.sola@biol.pmf.hr (I.Š.); eniksa@stud.biol.pmf.hr (E.N.); 2 Department of Pharmaceutical Botany, Faculty of Pharmacy and Biochemistry, University of Zagreb, A. Kovačića 1, 10000 Zagreb, Croatia; zeljan.males@pharma.unizg.hr

**Keywords:** antioxidant potential, antidiabetic potential, in vitro digestion, polyphenols, antihyperlipidemic potential

## Abstract

The present work evaluates the effect of casein, glucose, and olive oil on phytochemical bioaccessibility, antioxidant potential (DPPH and FRAP), antidiabetic potential (inhibition of amylase, α-glucosidase, and BSA glycation), and antihyperlipidemic potential (inhibition of lipase) of gingko standardized leaf extract in the form of tablets after in vitro digestion. Gingko extract formulations with protein, carbohydrates, and oil had high (>70%) in vitro bioaccessibility of quercetin, kaempferol, and isorhamnetin after each phases of digestion in comparison to moderate (35–70%) in vitro bioaccessibility from gingko water extract. Formulation with the highest in vitro bioaccessibility of the majority of the tested polyphenolic groups and terpene lactones after oral and intestinal phases was ginkgo with olive oil. High (>70%) antioxidant (DPPH and FRAP), antidiabetic (α-glucosidase and BSA glycation), and antihyperlipidemic potential were detected in almost all ginkgo formulations. Based on the results, we conclude that the in vitro bioaccessibility of individual compounds or groups of compounds depends on whether the tablets are taken with water or with foods (protein, carbohydrates, and oil).

## 1. Introduction

Ginkgo leaf extract is used as a dietary supplement. It is used in the treatment of disorders of the central nervous system, such as Alzheimer’s disease and dementia [1]. The main positive effects of *Ginkgo biloba* L. leaves are increased blood circulation and tissue oxygenation, antagonistic action against platelet-activating factor (PAF) receptors, prevention of cell damage by free radicals, protection of mitochondrial function during cellular stress, increase in memory and cognitive function (especially in the elderly), protection of nerve tissue, assistance in adaptation to stressors, modulation of vascular risk, reduction in anxiety. According to Eisvand et al. [2], ginkgo leaf extract has an impact on weight loss; antidiabetic, antihypertensive, and hypolipemic effects associated with metabolic syndrome (MetS) that increases the risk of cardiovascular disease. Good effects, on peripheral and cerebral blood flow, occur thanks to the chemical components of the extract, which inhibit platelet aggregation and increase the release of nitric oxide (NO) into the blood vessels. Therapeutic mechanisms of action are attributed to antioxidant properties and free radical scavenging properties. Thanks to its antioxidant properties, oxidative stress is also reduced, which is closely related to damage and the development of various diseases, including cardiovascular and neurodegenerative diseases as well as the appearance of cancer. In the case of antioxidant deficiency, concentrations of oxygen and nitrogen reactive species are often increased, which then lead to lipid peroxidation. Antioxidants from *G. biloba* leaf extract react with reactive species and thus inhibit lipid peroxidation, so the cytoprotective activity of this plant extract is attributed to antioxidant activity and the ability to increase the activity of endogenous enzymes that remove free radicals such as superoxide dismutase and catalase [1,2].

The typical daily dose is 120 to 240 mg of standardized ginkgo plant extract in a 50:1 ratio, with 24% flavonoid glycosides and about 6% terpenoids [1]. Such an extract can be in liquid form or as part of a tablet. So far, some research has been conducted to increase the oral bioavailability of flavonoids, ginkgolide, and bilobalide from ginkgo. Tang et al. [3] conducted a study on improving the oral bioavailability of active components from ginkgo since they are poorly soluble, resulting in lower bioavailability values. They conducted an in vivo study on a sample of six healthy dogs whose weight varied between 20 and 28 kg. They were given 800 mg tablets orally with and without the use of self-emulsifying drug delivery systems (SEDDS). SEDDS consists of oil, surfactant, and cosurfactant that exist as an emulsion within an aqueous medium. They took blood and plasma samples from which LC-ESI-MS analysis detected concentrations of ginkgolide and bilobalide. More positive results were obtained for the combination of ginkgo and SEDDS, since the concentrations of bilobalide, as well as ginkgolide A and B were 162.1; 154.6%, and 155.8% higher than pure tablets. As a conclusion, they stated that the combination with SEDDS promotes a more successful dissolution of these phytochemicals, and thus their bioavailability. Since the bioavailability of flavonoids in the brain is very low due to the brain’s blood–brain barrier and due to their low solubility in the lipophilic medium that prevents them from passing through biological membranes, new approaches based on nanotechnology and proliposomes are being developed, which include the addition of substances such as oil, sugar, or protein to increase bioavailability [4]. Phytochemicals’ bioavailability studies on animals and humans have limitation. They are expensive, time-consuming, and require special approvals for ethical reasons. So, in vitro digestive models represent a good alternative to test phytochemical release from the food matrix and give information about the in vitro bioaccessibility of phytochemicals and evaluate their profile change before absorption [5]. Olivera et al. [6] conducted a study that determined that the in vitro digestion model is a good method for studying antioxidant activity and providing insight into the in vitro bioaccessibility of phenolic substances from ginkgo leaf extract, as this model can easily and quickly simulate important physicochemical and biochemical conditions of the digestive tract. They also emphasize that the method does not include some aspects of the in vivo process, such as the activity of microorganisms along the digestive tract. Given their poor solubility and good permeability, triterpene and diterpene lactones have poor bioavailability [7]. Wang et al. [8] conducted a study in which they prepared tablets from ginkolide powder and soy phospholipids to improve the bioavailability of lactones. Soy phospholipid is an amphipathic molecule that binds to lactones and promotes their dissolution, and thus bioavailability in the in vivo system.

The aim of this study was to determine the in vitro bioaccessibility of ginkgo bioactive substances and antioxidant, antidiabetic, and antihyperlipidemic potential after application of ginkgo extract in combination with protein, carbohydrates, and oil in order to determine the effect of individual nutrients on the in vitro bioaccessibility of ginkgo bioactive substances. This research could give us information on how different food matrices/meals/formulations influence the in vitro bioaccessibility of ginkgo phytochemicals. For this purpose, the effects of protein (casein with a concentration of 40 g/L), carbohydrates (glucose with a concentration of 25 g/L), and oil (5% olive oil) on the in vitro bioaccessibility of polyphenols and the antioxidant, antidiabetic, and antihyperlipidemic potential of *G. biloba* extract under in vitro digestion conditions were investigated.

## 2. Results and Discussion

### 2.1. In Vitro Bioaccessibility of Polyphenols and Triterpene Lactones

Bioavailability includes digestion and absorption efficiency of certain constituents of food and drugs digested by oral administration [9]. Foods and drugs are mainly digested and absorbed in the small intestine after gastric digestion [9,10]. In vitro bioaccessibility of the total and individual polyphenols (total phenols (TP), flavonoids (TF), phenolic acids (TPA), hydroxycinnamic acids (THA), flavonols (TFLO), flavanols (TFLA), proanthocyanidins (TPAN), quercetin (Q), kaempferol (K), isoramnetin (IzoR), and total identified flavones (TiF) and triterpene lactones (triterpene lactones expressed in gingkolide A (TTL-A) and B (TTL-B) equivalents) of ginkgo water, casein (40 mg/mL), glucose (25 mg/mL), and olive oil (5%) formulations) after in vitro digestion is presented in Table 1.

The highest in vitro bioaccessibility of TP was from the ginkgo water formulation through all the stages of in vitro digestion (mouth, stomach, and small intestine). Ginkgo casein formulation also had the highest in vitro bioaccessibility of TP but only after the gastric phase of in vitro digestion. We hypothesize that low pH of the solution in the gastric phase of digestion has a positive effect on the bond between protein casein and polyphenols as protein dissociation occurs and the binding site is exposed to protein interaction with polyphenols through electrostatic interactions, which confirms Ozdal et al. [11]. The in vitro bioaccessibility of TF varied depending on the stage of digestion. So, the highest in vitro bioaccessibility of TF was from the ginkgo casein and olive oil formulation after the oral phase, ginkgo water and olive oil formulation after gastric phase, and from ginkgo casein formulation after the intestinal phase of in vitro digestion. Ginkgo casein formulation had the highest in vitro bioaccessibility of TPA after each of the stages of in vitro digestion (mouth, stomach, and small intestine). The highest in vitro bioaccessibility of THA was detected from the ginkgo glucose formulation after the oral and gastric phases, and from the ginkgo water and glucose formulation after the intestinal phase of in vitro digestion. The ginkgo olive oil formulation had the highest in vitro bioaccessibility of TFLO, TFLA, and TPAN after each of the in vitro digestion phases (mouth, stomach, and small intestine). According to Haratifar and Corredig [12], 60% of green tea flavanols are degraded after the intestinal phase of digestion. The highest TTL, expressed as equivalents of ginkgolides A and B, was detected in the ginkgo olive oil formulation after the oral and intestinal phases, and in ginkgo water formulation after the gastric phase of in vitro digestion. Since most drugs are digested and absorbed in the small intestine, we suggest that the best gingko formulation for TP digestion and absorption is that with water, for TPA formulation with water and glucose, for TF and TPA with casein, and for TFLO, TFLA, TPAN, and TTL with olive oil. The in vivo bioavailability of polyphenols [13] and triterpene lactones [14] is very low. The highest absorption rate after the intestinal phase of digestion from the flavonoid group was recorded for isoflavones, catechins, flavanones, and quercetin glucosides and the lowest for proanthocyanidins, catechins, and anthocyanins [13]. According to the percentage classification of Vujčić Bok et al. [15], all of the tested gingko formulations had high (>70%) in vitro bioaccessibility of TP, TF, THA, TFLO, TFLA, and TTL after the oral phase, high (>70%) in vitro bioaccessibility of TF, TPA, TFLO, and TTL after the gastric phase, and high (>70%) in vitro bioaccessibility of TF, THA, TFLO, and TTL after the intestinal phase of in vitro digestion. Moderate in vitro bioaccessibility (35–70%) was recorded for TPA after the oral phase from the ginkgo water and ginkgo glucose formulation. After the gastric phase of digestion, moderate in vitro bioaccessibility was measured for TP from the formulation of ginkgo glucose and ginkgo olive oil, for THA from the formulation of ginkgo casein, for TFLA from the formulations of ginkgo water and ginkgo glucose, and for TPAN from the formulations of ginkgo water and ginkgo casein. Moderate in vitro bioaccessibility of TP after the intestinal phase was recorded from the ginkgo casein, ginkgo glucose, and ginkgo olive oil formulations, for TPA and TPAN from the ginkgo water and ginkgo glucose formulations, for TFLA from the ginkgo water, ginkgo casein and ginkgo glucose formulations, and for TTL from the ginkgo glucose formulation. Weak in vitro bioaccessibility (<35%) was measured for TPAN only from ginkgo water and ginkgo glucose formulations after the intestinal phase of digestion. The high in vitro bioaccessibility of the tested polyphenolic and terpene groups after almost every phase of in vitro digestion compared to the results of other authors [6] may be due to the fact that we used standardized ginkgo leaf extracts in tablets and did not centrifuge the formulations prior to in vitro digestion. This means that after each phase of digestion, more polyphenols and triterpene lactones could be released from the grounded gingko tablets. After the oral phase, the highest in vitro bioaccessibility of the most polyphenolic groups was detected from the ginkgo water formulation for TP, the ginkgo glucose formulation for THA, and the gingko oil formulation for TFLO, TFLA, and TPAN. The highest in vitro bioaccessibility of TF was detected from the ginkgo oil formulation after the gastric phase of digestion. The in vitro bioaccessibility of TTL was the highest from the ginkgo water formulation after the gastric phase of digestion. After the intestinal phase of digestion, the highest in vitro bioaccessibility of TPA was detected from the ginkgo casein formulation.

The highest in vitro bioaccessibility of the total identified flavonoids (TiF) was from the ginkgo glucose formulation after each of the in vitro digestion phases. Gingko formulations with the addition of protein, carbohydrates, and oil had significantly higher concentrations of Q, K, and IzoR after the gastric and intestinal phases compared to gingko water formulation. Additionally, all the formulations with the addition of protein, carbohydrates, and oil had high (>70%) in vitro bioaccessibility of individual compounds (Q, K, and IzoR) after each phase of digestion compared to the gingko water formulation with moderate (35–70%) in vitro bioaccessibility after most of the in vitro digestion phases. Consequently, casein, glucose, and olive oil have positive effect on the in vitro bioaccessibility of total and individual flavonols, which together with terpene lactones (ginkgolides A and B, bilobalide) represent the main components of ginkgo leaf extract. Higher concentrations of these flavonols and terpene lactones do not necessarily lead to increased bioavailability in the in vivo system.

According to Kardum and Glibetic [16], olive oil is the best source of oleic acid, which, among other positive effects, has a stabilizing effect on phenolic compounds. Consuming foods rich in lipids might increase the absorption of flavonoids, especially Q and IsoR [17]. The combination of fat and phenolic compounds stimulates the secretion of bile, which promotes better absorption and micellarization of individual phenolic compounds in the intestine. The results from both groups of authors correspond to our results, which show a significant increase in the in vitro bioaccessibility of individual (Q, K, and IzoR) and total flavonols (TFLO and TiF) from gingko olive oil formulation in comparison to gingko water formulation. Glycosylated quercetin and kaempferol have higher bioavailability in humans than their aglycone forms [18]. Ginkgo formulation with glucose after almost each of the digestion phases had high in vitro bioaccessibility of individual (Q, K, and IzoR) and total (TFLO and TiF) flavonols. The formulation with carbohydrates could increase the in vitro bioaccessibility by changing the viscosity and decreasing the water activity [19]. Kaempferol has greater chemical stability than quercetin as it has fewer hydroxyl groups. In our study, moderate in vitro bioaccessibility of Q after the oral phase was detected compared to high in vitro bioaccessibility of K from the ginkgo water formulation. After gastric and intestinal digestion, both Q and K had moderate in vitro bioaccessibility. In vitro bioaccessibility results have some limitations because long-term stability of bioactive compounds during digestion and the potential effects of gut microbiota were not tested. Choi et al. [20], in their in vivo study on antibacterial-treated mice, investigated the effects of oral intake of microbiota and ginkgo leaf extract rich with terpene lactones (bilobalide, ginkgolide A, ginkgolide B, and ginkgolide C) and flavonols (isorhamnetin, kaempferol, and quercetin) and recorded the positive influence of microbiota on the isorhamnetin bioavailability. 

### 2.2. Antioxidant Activity

**Figure 1:**
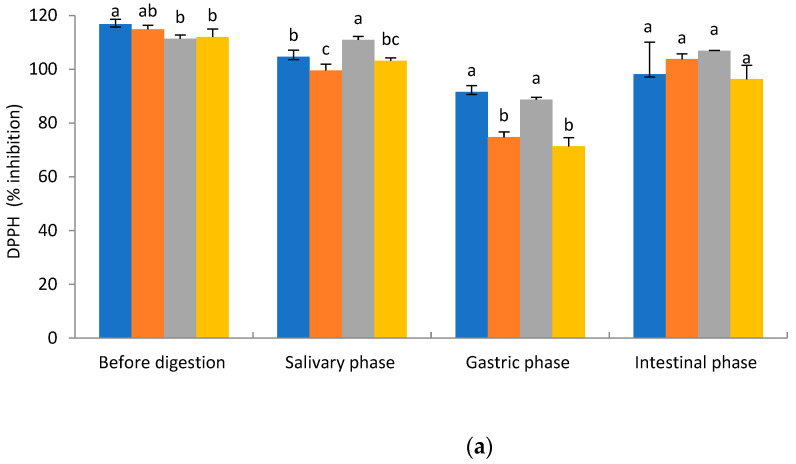

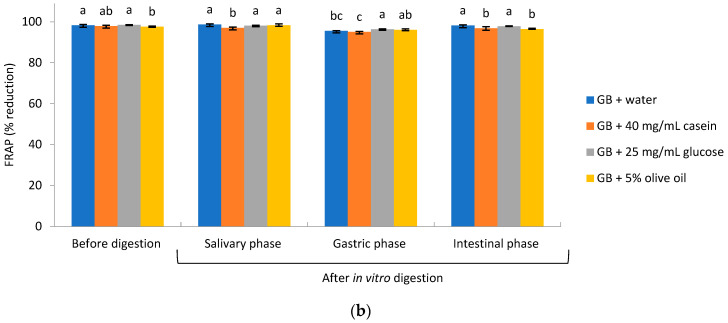
Inhibition percentage of (**a**) 2,2-diphenyl-1-picrylhydrazyl radical (DPPH) and (**b**) reduction percentage of Fe^3+^ measured by ferric reducing antioxidant power (FRAP) method in relation to the Trolox for each phase before and after in vitro digestion for all ginkgo formulation (GB). Data represent mean values of three replicas ± standard deviation. Statistically significantly different values were denoted by different letters (one-way ANOVA, Duncan test, *p* < 0.05). Statistical analysis was performed separately for each phase before and after in vitro digestion.

The antioxidant activity was assessed using two standard colorimetric tests: FRAP (test based on the transfer of one electron) and DPPH (test based on the transfer of both a hydrogen atom and an electron). Both tests do not fully replicate physiological conditions, but they are fast, simple, and cost-effective [21]. The percentage of DPPH radical inhibition and reduction percentage of Fe^3+^, in relation to the Trolox formulations, of ginkgo water, ginkgo casein (40 mg/mL), ginkgo glucose (25 mg/mL), and ginkgo olive oil (5%) are presented in Figure 1. After the salivary phase, the highest antioxidant activity measured by the DPPH method showed the ginkgo glucose formulation. The ginkgo formulation with water and glucose had the highest antioxidant activity measured by the DPPH method compared to all other formulations tested. After intestinal digestion, no significant differences in antioxidant activity were observed between ginkgo formulations with the DPPH method. With the FRAP method (Figure 1b), the highest antioxidant activity was recorded before digestion, and after salivary and intestinal digestion for the ginkgo water formulation and the ginkgo glucose formulation. After gastric digestion, the highest antioxidant activity was observed with the FRAP method for the ginkgo glucose formulation. According to Vujčić et al. [22], percentages of gradation of antioxidant activity, all ginkgo formulations had high (>70%) antioxidant activity measured by the DPPH and FRAP method before and after in vitro digestion. Polyphenols are a group of specialized metabolites that have pronounced antioxidant properties [23,24]. In our study, we reported high bioavailability (>70%) of the majority of the individual flavonols tested, total identified flavonoids (TiF), and total polyphenolic groups after almost every phase of in vitro digestion. The high bioavailability of ginkgo polyphenols could be responsible for the strong antioxidant activity of the ginkgo extract tested. Quercetin (Q), kaempferol (K), and isorhamnetin (IsoR) were identified as the main flavonoids in our ginkgo formulation. All identified flavonoids are strong antioxidants, according to the literature [17,18,25].

### 2.3. In Vitro Digestive Enzyme Inhibitory Activity and BSA Glycation

**Figure 2:**
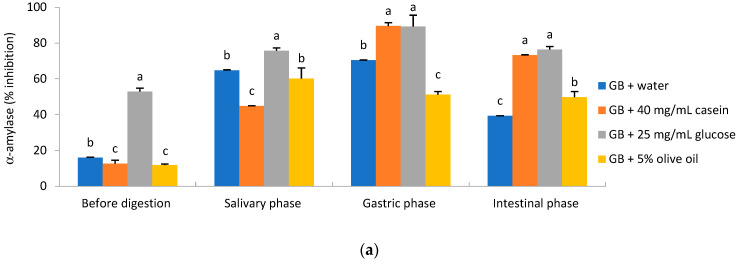

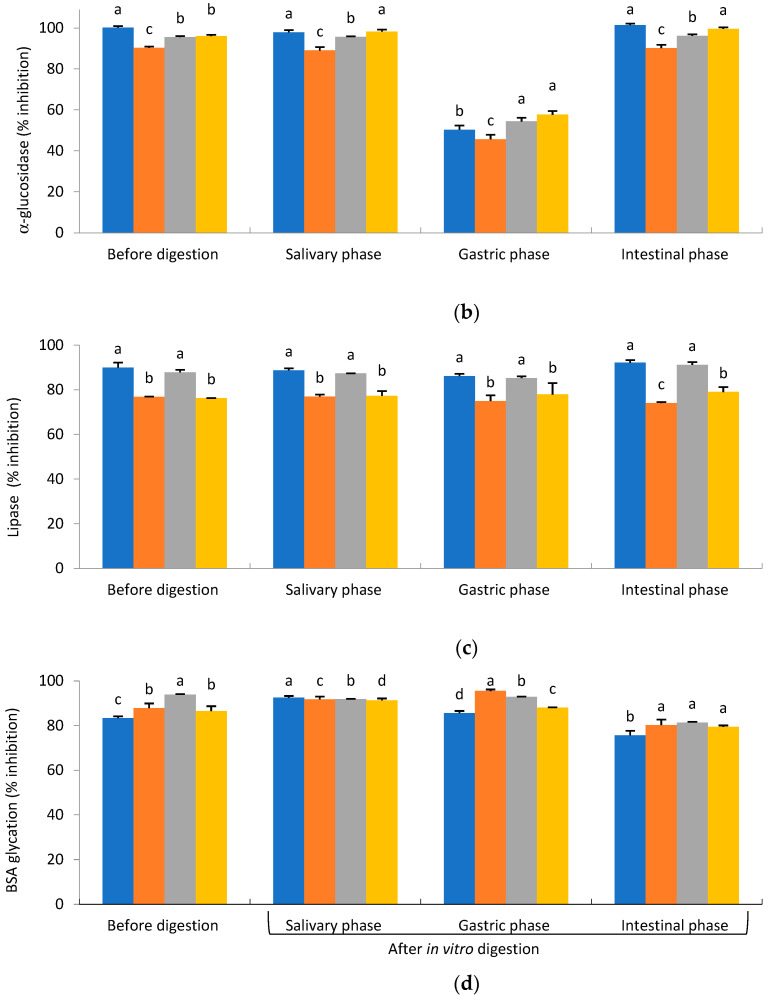
In vitro inhibitory activities of ginkgo samples (GB) in relation towards digestive enzymes: (**a**) α-amylase, (**b**) α-glucosidase, (**c**) lipase, and (**d**) glycation of bovine serum albumin (BSA). Data represent mean values of three replicas ± standard deviation. Statistically significantly different values were denoted by different letters (one-way ANOVA, Duncan test, *p* < 0.05). Statistical analysis was performed separately for each phase before and after in vitro digestion.

Among antioxidants, polyphenols are known as good inhibitors of enzyme involved in the glucose (α-amylase and α-glucosidases) and lipids digestion (lipase). Inhibition of these enzymes is of great importance in patients with type-2 diabetes mellitus (T2DM), which is characterized by postprandial hyperglycemia, and patients with obesity, which is characterized by hyperlipidemia. Medications such as acarbose are effective in lowering postprandial hyperglycemia, but acarbose has many unpleasant side effects such as indigestion, bloating, and diarrhea due to the fermentation of undigested sugars. Therefore, there is a need for natural preparations for the prevention and treatment of T2DM [26,27,28]. The highest potential to inhibit α-amylase was detected for the formulation of ginkgo with glucose before and after each of digestion phases (Figure 2a). Additional oral glucose ingestion is not recommended in patients with diabetes, therefore, the best ginkgo formulation with the highest inhibition of α-amylase after the gastric and intestinal phases of digestion was that with casein or glucose (Figure 2a). Inhibition of the α-amylase enzyme results in reduced carbohydrate degradation in the upper part of the intestine and slows the absorption of starch and disaccharides ingested orally in the mouth and in the lower part of the small intestine. Compounds such as kaempferol and quercetin are known to be able to inhibit α-amylase [29]. In our study, we detected an increase in the bioavailability of Q and K from the gingko combined with casein, glucose, or olive oil compared to the gingko water formulation (Table 1). The highest potential to inhibit α-glucosidase was detected for the ginkgo/water formulation before digestion, the ginkgo/water and ginkgo/glucose formulation after oral and intestinal digestion, and the ginkgo/olive oil and ginkgo/glucose formulation after the gastric phase of digestion (Figure 2b). α-glucosidase is located in the epithelium of the small intestine [30], which is why the results of the intestinal phase are the most relevant. Depending on the percentage of inhibition versus positive control acarbose, antidiabetic activity can be classified as weak (<35%), moderate (35–70%), and strong (70–100%) [31]. Consequently, the ginkgo casein and ginkgo glucose formulation had strong antidiabetic activity, according to α-amylase inhibition, and the ginkgo water and ginkgo olive oil formulation had moderate antidiabetic activity after the intestinal phase. Strong antidiabetic activity, based on the inhibition of α-glucosidase, was detected for all formulations of ginkgo after the intestinal phase of digestion. Our results on the inhibition of digestive enzymes (α-amylase and α-glucosidases) by ginkgo extracts are in accordance with the results of other authors [32,33,34]. Therefore, the ginkgo formulation with strong inhibition of α-amylase and α-glucosidase could be used as a potential natural hypoglycemic agent with fewer side effects than medications such as acarbose. According to Feng et al. [34], the flavonoid fraction of ginkgo extract is the most responsible for antidiabetic activity. We detected a high bioaccessibility of TF, TiF, Q, K, and IzoR from almost all of the ginkgo formulations after the intestinal phase of in vitro digestion, which confirms the above claim. Protein glycation is known as the Maillard reaction between protein molecules and glucose molecules. It is a non-enzymatic reaction of reducing sugars and proteins that includes steps in the formation of Schiff base, Amadori products, and advanced glycation end products that are responsible for a number of disorders such as vasculopathy, retinopathy, neuropathy, cataracts, and chronic kidney disease. Given the pathological consequences of glycation, it is important to identify protein glycation inhibitors that could prevent diabetes-related complications [35]. According to Van der Lugt et al. [36], glycation products are formed during the intestinal phase of digestion. Therefore, the most relevant are the results of intestinal digestion in which the ginkgo casein, ginkgo glucose, and ginkgo olive oil formulations had statistically higher inhibition of BSA glycation compared to the ginkgo water formulation. All ginkgo formulations had high (>70%) inhibition of BSA glycation. Adisakwattana et al. [32] reported moderate inhibition of BSA glycation. The higher percentage in our study might be because we used standardized ginkgo leaf extracts instead of dry ginkgo leaf. Pancreatic lipase is involved in the breakdown of fats including triacylglycerides and phospholipids that play an essential role in lipid metabolism. Obesity is highly correlated with cardiovascular disease and hyperglycemia. Orlistat is a drug available to treat obesity because it is a very good pancreatic lipase inhibitor that reduces lipid digestion and absorption at the peripheral level. However, it has many unpleasant gastrointestinal side effects. Therefore, it is necessary to find natural preparations with the same or similar action as orlistat to reduce the risk of obesity, type 2 diabetes mellitus, and cardiovascular disease [27]. Statistically higher lipase inhibition potential was recorded for the ginkgo water and ginkgo glucose formulations before and after each of the digestion phase. All ginkgo formulations had high lipase inhibition potential (>70%), indicating that all formulations could have a positive influence on weight loss and reduce the risk of cardiovascular disease and hyperglycemia. According to Shahidi and Ambigaipalan [37], the consumption of foods high in phenolic compounds is associated with a reduced risk of developing obesity. Very high bioavailability of TF was detected after every phase of in vitro digestion. This suggests that TF could be responsible for the high inhibition of the enzyme lipase.

### 2.4. Principal Component Analysis (PCA)

**Figure 3:**
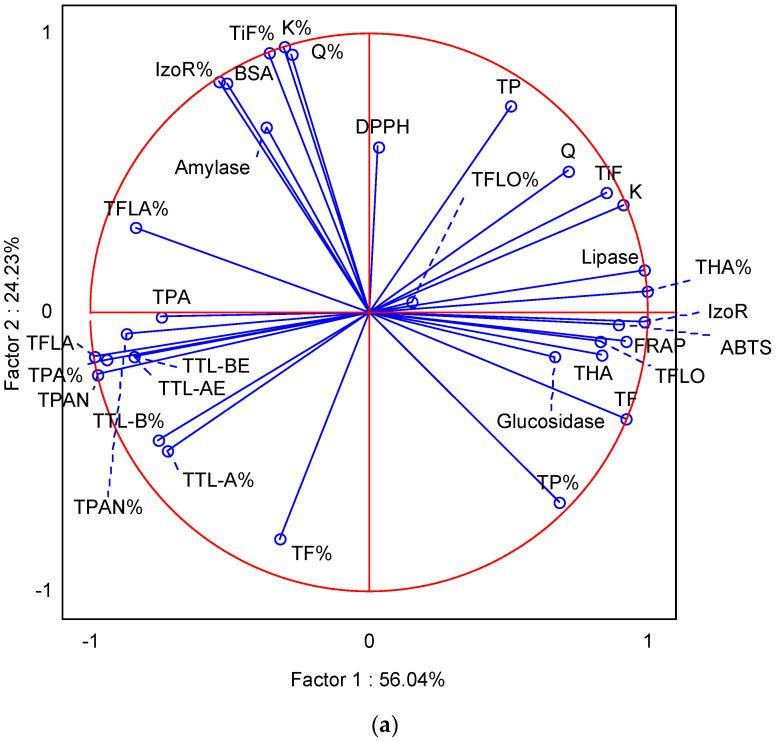

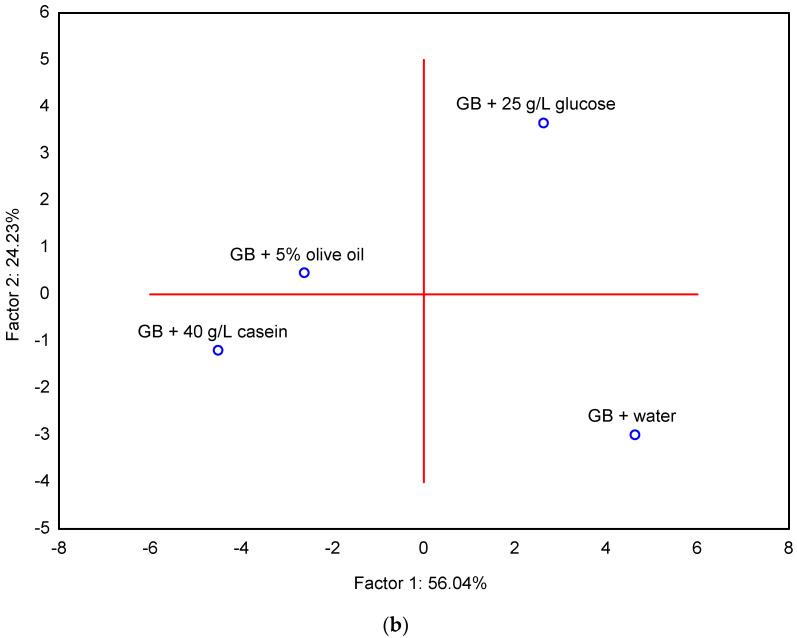
Principal component analysis diagram (PCA): (**a**) performed on the correlation matrix of average values of phytochemical composition, normalized (%) bioaccessibility of total phenols (TP), flavonoids (TF), phenolic acids (TPA), hydroxycinnamic acids (THA), flavonols (TFLO), flavanols (TFLA), proanthocyanidins (TPAN), triterpene lactones expressed in equivalents of gingkolide A (TTL-AE) and B (TTL-BE), quercetin (Q), kaempferol (K), isoramnetin (IzoR), and total identified flavones (TiF); antioxidant methods (DPPH and FRAP) and inhibition of enzymes amylase, glucosidase and lipase, and glycation of bovine serum albumin (BSA) for the intestinal phase of in vitro digestion; and (**b**) ginkgo samples (GB) in combination with water, casein, glucose, and olive oil after the intestinal phase of in vitro digestion.

The PCA is a statistical method used to gain insight into the relationship between the phytochemical composition of the plant and the biological activity (antioxidant and antidiabetic) in plant extracts and formulations [15,38]. In order to gain insight into the similarities and differences between the formulations of ginkgo and the measured parameters, we analyzed the main components with standardized in vitro bioaccessibility data, total and individual phytochemical measurement data, and antioxidant, antidiabetic, and antihyperlipidemic potential data after the intestinal phase of in vitro digestion (Figure 3a,b). The first factor and the second factor described 55.33% and 24.98% of the variance after gastrointestinal digestion. In the upper right square of the PCA diagram, there was a ginkgo water formulation with TP bioaccessibility (%), TF, THA, TFL, TFLO, and IzoR content, as well as inhibition of glucosidase and antioxidant potential measured by the FRAP method, and in the upper left square, there was ginkgo casein formulation with TF, TPAN, TFLA, TPA, TTL-A and TTL-B (%), TTL-A, TTL-B, TPAN, and TPA content, in the lower right square, there was a ginkgo glucose formulation with bioaccessibility of TFLO and THA (%), TP, Q, K, and TiF content, as well as inhibition of lipase and antioxidant potential measured by the DPPH method, and in the lower left square, there was a ginkgo olive oil formulation with bioaccessibility of TFLA, TiF, IzoR, Q, and K (%), as well as inhibition of amylase and BSA glycation. After the intestinal phase of in vitro digestion, PCA gave a clear separation of the ginkgo water formulation and the gingko casein formulation with the highest distance, suggesting significant difference between these formulations (Figure 3b). We could also observe a high distance between the ginkgo water and the gingko oil formulation, and between the ginkgo water and the ginkgo glucose formulation. The ginkgo formulations with olive oil and casein were the least distant, suggesting higher similarity between the two samples. Antioxidant potential could mainly be associated with ginkgo water and ginkgo glucose formulations because among them, the smallest distance on the PCA diagram was observed. The same formulations can be mostly associated with inhibition of lipase. This indicates a positive effect on weight loss that reduces the risk of developing hyperglycemia and the development of complex chronic type 2 diabetes. Antidiabetic activity can mostly be associated with the formulations of ginkgo with olive oil, casein, and glucose. Depending on the position of the antioxidant and antihyperlipidemic results in the PCA diagram and their mutual distance, we conclude that antioxidant and antidiabetic potential are associated with the content and bioaccessibility of most of the individual and total polyphenols, which is consistent with the literature [15,38,39,40,41]. Triterpene lactones are far removed from the results on antioxidant and antidiabetic potential, suggesting that they lack significant antioxidant and antidiabetic activity. According to the literature, triterpene lactones are attributed mostly to the antiplatelet and neuroprotective effects [42].

## 3. Materials and Methods

### 3.1. Materials and Preparation of Ginkgo Formulations

The flavonoid and phenolic acid standards were of HPLC grade and purchased from Sigma-Aldrich GmbH (Taufkirchen, Germany) or Extra synthese (Genay, France). All other chemicals and reagents were supplied by Sigma-Aldrich GmbH (Taufkirchen, Germany) or Kemika (Zagreb, Croatia). The chemicals and reagents were of analytical grade.

Standardized *Ginkgo biloba* L. leaf extract tablets (brand: M. E. V. Feller, Lot. No. 1789005, origin: United Kingdom) were purchased in a local pharmacy (Zagreb, Croatia). Two ginkgo tablets were grounded (80 mg of standardized ginkgo leaf extracts per tablet) and overflowed with 4 mL of deionized water, 40 mg/mL casein (Sigma Aldrich GmbH, Taufkirchen, Germany) water solution, 25 mg/mL glucose (Kemika, Zagreb, Croatia) water solution, and 5% olive oil (Zvijezda plus d.o.o., Zagreb, Croatia) water solution and vortex for 15 s. Immediately after the preparation of ginkgo water, ginkgo casein (40 mg/mL), ginkgo glucose (25 mg/mL), and ginkgo olive oil (5%) formulations, in vitro digestion of the samples was performed.

### 3.2. In Vitro Digestion

The in vitro digestion model was performed according to Rusak et al. [31]. First, the volume of 0.3 mL of ginkgo water, casein (40 mg/mL), glucose (25 mg/mL), and olive oil (5%) formulations was mixed with the same volume of 20 mM phosphate buffer pH 7.0. The salivary phase of digestion was initialized with 10 μL of amylase (0.48 mg/mL in 20 mM phosphate buffer pH 7.0) and incubated for 5 min at 37 °C in a shaking water bath at 150 rpm. To simulate the stomach digestion, 0,4 mL of porcine pepsin solution (3 mg/mL in 0.1 M HCl) was added and acidified with 0.5 M HCl (pH 2.0). The samples were incubated in a shaking water bath SW22 (Julabo, Seelbach, Germany) for 1 h at 37 °C at 150 rpm. The upper intestinal phase of digestion was first mimicked by adding sodium bicarbonate (1 M NaHCO_3_) to adjust the pH to 5.3. After pH adjustment, volume of 0.9 mL of pancreatic juices (2.4 mg bile acids/mL, 0.2 mg porcine lipase/mL, and 0.4 mg pancreatin/mL in 20 mM phosphate buffer pH 7.0) was added. The final total volume of each intestinal phase sample was brought to 2 mL with 20 mM phosphate buffer (pH 7.0). The final pH was also adjusted to 7.0 with 1 M NaOH. The samples were then incubated for 2 h at 37 °C in a shaking water bath at 150 rpm. The final volume of each sample, both before and after digestion, was brought to 2 mL with 20 mM phosphate buffer (pH 7.0). The samples were centrifuged (Hettich MIKRO 220R; Andreas Hettich GmbH & Co., Tuttlingen, Germany) at 11,000 rpm for 10 min at 4 °C and the supernatants were stored at −20 °C until spectrophotometric and HPLC analyses. The in vitro digestion was carried out in triplicates. Each digestion stage contained samples with ginkgo leaf extract, samples with extract and glucose, samples with extract and casein, samples with extract, and olive oil and blanks. Blanks were distilled water samples, water glucose samples, water casein samples, and water olive oil samples. All these samples (before digestion and after salivary, gastric, and intestinal phases of in vitro digestion) were used for the spectrophotometric and HPLC analyses.

### 3.3. Spectrophotometric Phytochemical Analysis

The total polyphenols (TP) of all formulations were determined with Folin–Ciocalteau reagent according to Zhishen et al. [43]. A volume of 2 μL of the ginkgo test solution was diluted with 158 μL of deionized water and then 10 μL of Foline–Ciocalteau reagent was added. Subsequently, 30 μL Na_2_CO_3_ (1.88 M) was added, and the mixture was incubated for 30 min at 45 °C. The absorbance of the mixture was measured at 740 nm. The TP content was calculated from the calibration curve and expressed as gallic acid equivalents (mg GAE/g).

The content of total flavonoids (TF) of all formulations was determined with AlCl_3_ according to the method described by Zhishen et al. [43]. To dilute ginkgo solution (20 μL in 80 μL of dH_2_O) volume of 6 μL NaNO_2_ (5%) was added. After 5 min of incubation, volume of 6 μL AlCl_3_ (10%) was added and the mixture was incubated at room temperature for an additional 6 min. Subsequently, 40 μL NaOH (1 M) and distilled water were added to the final volume of 200 μL. The absorbance of the reaction mixture was read at 520 nm. The TF content was calculated from the calibration curve and expressed as quercetin equivalents (mg QE/g).

The content of phenolic acids (TPA) in all formulations was determined according to European Pharmacopoeia [44]. A volume of 40 μL of ginkgo solution was mixed with 80 μL HCl (0.5 M). In the solution, 80 μL of freshly prepared reagent (1 g NaNO_2_ i 1,17 g Na_2_MoO_4_ × 2H_2_O in 10 mL of deionized water) was added. Subsequently, 80 μL NaOH 8.5% (*w*/*w*) and distilled water were added to the final volume of 400 μL. The absorbance of the reaction mixture was read at 492 nm. The TPA content was calculated from the calibration curve and expressed as caffeic acid equivalents (mg CAE/g).

The total content of hydroxycinnamic acids (THA) and total flavonols (TFLO) of the gingko samples were measured according to the method of Howard et al. [45] using caffeic acid and quercetin as standards. A volume of 50 μL of the extract (3 g/L) was mixed with 50 μL HCl (1 g/L in ethanol) and 0.91 mL of HCl (2 g/L). The absorbance of the solution was read at 320 and 360 nm, respectively. The THA and TFL contents were calculated from the corresponding calibration curves and expressed as caffeic acid (mg CAE/g) and quercetin equivalents (mg QAE/g), respectively.

The total flavanol (TFLA) content was determined using *p*-dimethylaminocinnamaldehyde (DMACA) according to Kusznierewicz et al. [46]. A volume of 100 μL of the ginkgo tested solution was mixed with 150 μL of DMACA solution (0.1% in 1 M HCl in MeOH). After 10 min of incubation at room temperature, the absorbance at 595 nm was measured. The TFL content was calculated from the calibration curve and expressed as catechin equivalents (mg CE/g).

The total proanthocyanidin (TPA) content was measured according to the vanillin-HCl assay described by Sun et al. [47]. The solution of vanillin (4% in methanol) was mixed with 25 μL of the ginkgo samples and 75 μL of HCl (conc.). After 15 min of incubation, the absorbance of reaction mixture was read at 492 nm. TPA content was calculated from the calibration curve and expressed as catechin equivalents (mg CE/g).

The determination of terpene trilactones (TTL) was determined according to Su et al. [42] at a wavelength of 520 nm. A volume of 40 µL of diluted gingko sample in 96% ethanol (1:9), and 16 µL of hydroxylamine solution containing 13.9% solution of hydroxylammonium chloride and sodium hydroxide (3.5 M) was added in a volume ratio of 1:2. The solution was then incubated for 5 min at 25 °C. After incubation, 16 µL HCl solution (3 M), 8 µL FeCl_3_ solution (6%), and 200 µL ethanol (70%) were added. The TTL content was calculated from the calibration curve and expressed as ginkgolides A and B equivalents (mg GIN-AE/g, mg GIN-BE/g).

### 3.4. RP-HPLC Analysis of Polyphenol Compounds

Before HPLC analysis, ginkgo samples were purified from protein according to Kendrick Labs Inc. protocol in 96% ethanol (1:9), incubated for 2 h at −80 °C, and centrifuged for 15 min at 13,000 rpm. The purified ginkgo supernatants were then hydrolyzed with 1.2 M HCl as described in Šola et al. [38]. The solutions were centrifuged three times (13,000 rpm, 5 min) and the supernatants stored at −20 °C until analysis.

Qualitative and quantitative RP-HPLC analysis was performed using the Agilent 1100 Series system equipped with a quaternary pump, multiwave UV/Vis detector, autosampler, fraction collector, Zorbax SB C-18 analytical guard column (12.5 × 4.6 mm, 5 µm particle size), and Poroshell 120 EC-C18 column (100 × 4.6 mm, 4 µm particle size) (Agilent Technologies, Waldbronn, Germany). Mobile phase A was 0.2% acetic acid and mobile phase B was 0.2% acetic acid and 80% methanol and the solvent gradient profile as in Šola et al. [39]. The flow rate was 1 mL/min and the injected volume of the sample was 20 μL. For quantification, the multiwave UV/vis detector was set at 360 nm for the determination of quercetin, kempferol, and isorhamnetin. Quercetin (Q), kaempferol (K), and isorhamnetin (IsoR) were characterized according to their retention times and UV spectra compared to commercial standards. For quantitative analyses, calibration curves were obtained by injection of 5 known concentrations (in the range of 1–250 µg/mL) of the 96% EtOH standard mixed solution in triplicate. The injection volume was 5 µL. Phenolics quantification was made by integration of peak areas with reference to calibration curves made using known amounts of available pure standard compounds. The results are expressed as mg/L. The obtained chromatograms are shown in the Figure S1.

### 3.5. Antioxidant Activity Assays

The free radical scavenging activity was measured using a stable free radical 1,1-diphenyl-2-picrylhydrazyl (DPPH) following the method of Radić Brkanac et al. [48]. Briefly, 10 μL of tested ginkgo solution was added to 190 μL of freshly prepared ethanolic DPPH solution (0.1 mM) and incubated in the dark for 30 min at room temperature. The decrease in absorbance was measured at 520 nm. The radical scavenging activity was calculated from the equation: % inhibition = [(A_0_ − A_t_)/A_0_] × 100, where A_0_ was the absorbance of the control (blank, without tested solution) and A_t_ was the absorbance in the presence of the tested solution. Trolox was used as a positive control.

The ferric reducing antioxidant power (FRAP) assay was performed according to the original method of Benzie and Strain [49]. The ginkgo-tested solution (10 μL) was mixed with the freshly prepared FRAP reagent (190 μL) and the absorbance was read at 595 nm after the reaction time of 4 min. The percent of Fe^3+^—TPTZ reduction was calculated using the formula: % reduction = [(A_t_ − A_0_)/A_t_] × 100, where A_0_ was the absorbance of the control (blank, without tested solution) and A_t_ was the absorbance in the presence of the tested solution. Trolox was used as a positive control.

### 3.6. Enzyme Inhibitory Activity Assay and BSA Glycation

The antidiabetic properties of all formulations through inhibition of α-amylase and α-glucosidase were also tested using the preincubation methods as we previously described [31,38].

For α-amylase inhibitory activity, gingko formulations (20 μL) were mixed with 20 μL α-amylase from human saliva (5 unit/mL solution in ice cold distilled water) and 40 μL of 20 mM phosphate buffered saline (pH 6.9) and preincubated for 15 min at 37 °C. A volume of 20 μL of potato starch (1% *w*/*v* in 20 mM phosphate buffered saline pH 6.9) was added after preincubation. The final concentration of all the formulations used was 6 mg/mL and α-amylase was 1 unit/mL. After 15 min of incubation at 37 °C, 50 μL of dinitrosalicylic acid reagent was added and incubated at 85 °C for 15 min. The volume of 450 μL of distilled water was mixed with the tested solution and the absorbance was measured at 545 nm. α-glucosidase inhibitory activity method included mixing gingko formulations (10 μL) with 10 μL α-glucosidase (1.5 U/mL in phosphate buffer) and 130 μL phosphate buffer (50 mM, pH 6.5) and then preincubating for 15 min at 37 °C. A volume of 50 μL *p*-nitrophenyl-α-D-glucopyranoside (1 mM in phosphate buffer) was reincubated for 5 min at 37 °C. The enzyme reaction activity was terminated by adding of 50 μL Na_2_CO_3_ (0.1 M, pH 10). The absorbance was measured at 405 nm. Appropriate blanks and controls were carried out for both methods. Enzyme inhibitory activity was calculated from the equation % inhibition = 100 − [(A_t_ − A_tb_/A_c_ − A_cb_) × 100], where A_t_ was the absorbance of the test (with enzyme), A_tb_ was the absorbance of the test blank (without enzyme), A_c_ was the absorbance of the control (with enzyme), and A_cb_ was the absorbance of the control blank (without enzyme). Acarbose was used as a positive control for both methods.

The pancreatic lipase inhibition assay was performed according to the method described by Spinola et al. [28] at a wavelength of 405 nm. A volume of 40 µL of the ginkgo sample was mixed with 20 µL of the substrate solution *p*-NPB (10 M in ethanol 96%) and 40 µL of enzyme (2.5 g/L in 0.1 M phosphate buffer, pH = 8.0). The resulting mixture was incubated for 20 min at 37 °C. As a positive control, we used a solution of orlistat (6 g/L in ethanol). The inhibition activity of the lipase was calculated according to following equation: % inhibition = 100 − [(A_t_ − A_tb_/A_c_ − A_cb_) × 100], where A_t_ was the absorbance of the test (with enzyme), A_tb_ was the absorbance of the test blank (without enzyme), A_c_ was the absorbance of the control (with enzyme), and A_cb_ was the absorbance of the control blank (without enzyme).

The BSA glycation inhibition assay we performed according to the method of Spinola et al. [27]. Fluorescence was measured in dark microwell plates of Nunc F96. A volume of 100 µL of BSA solution (10 g/L) was mixed with 100 µL of fructose solution (0.5 M) and 40 µL of the ginkgo sample. The mixture was incubated for 24 h at 37 °C and then fluorescence was measured at an excitation wavelength of 405 nm and an emission wavelength of 460 nm. As a positive control, we used a catechin solution (6 g/L). BSA glycation inhibition was calculated according to the equation:

% inhibition = [(F_t_ − F_o_)/F_t_] × 100

where the symbol F_t_ represents the fluorescence of the tested sample and F_o_ the fluorescence of the blank.

All absorbance and fluorescence measurements were performed with Fluostar Optima microplate reader (BMG Labtech GmbH, Offenburg, Germany).

### 3.7. In Vitro Bioaccessibility of Ginkgo Phytochemicals

The bioaccessibility of total and individual bioactive compound (BC) was calculated from the following equations: bioaccessibility in salivary phase (%) = 100 × (BC salivary phase/BC before digestion), bioaccessibility in gastric phase (%) = 100 × (BC gastric phase/BC before digestion), and bioaccessibility in intestinal phase (%) = 100 × (BC intestinal phase/BC before digestion), where BC salivary phase, BC gastric phase, and BC intestinal phase corresponded to the total or individual bioactive compound concentration in salivary, gastric, and intestinal phases, and BC before digestion was the total or individual bioactive compound concentration before in vitro digestion.

### 3.8. Statistical Analysis

All results were evaluated using the Statistica 14.0.0.15 software package (TIBCO Software Inc., Santa Clara, CA, USA). RP-HPLC and results from spectrophotometric determination of phytochemical, antioxidant, and antidiabetic assays were subjected to one-way analysis of variance (ANOVA) for comparison of means, and significant differences were calculated according to Duncan’s multiple range test. Data are presented as mean ± standard deviations (SD). A principle component analysis (PCA) between phytochemicals, antioxidant, and antidiabetic activity was performed. Data were considered statistically significant at *p* < 0.05.

## 4. Conclusions

Terpene lactones are the most responsible for antiplatelet and neuroprotective activity in ginkgo leaf extracts [1]. We detected the highest in vitro bioaccessibility of terpene lactones in the intestinal phase of digestion in ginkgo olive oil and casein formulation. Therefore, consumption of ginkgo tablets with casein and olive oil could have a positive effect on antiplatelet and neuroprotective activity. The high in vitro bioaccessibility of quercetin, kaempferol, and isorhamnetin in all phases of in vitro digestion was detected in the formulation of gingko protein (casein), carbohydrates (glucose), and oil (olive oil) compared to the moderate in vitro bioaccessibility of the formulation of gingko water. Therefore, consumption of ginkgo tablets with protein, carbohydrate, and oil could have positive effects on anti-inflammatory, antimicrobial, radical-scavenging, and immune-modulatory activities. High-antioxidant (DPPH and FRAP), antidiabetic (α-glucosidase and BSA glycation), and antihyperlipidemic potential were detected in almost all ginkgo formulations. Ginkgo extract in combination with oil shows the best results due to its hydrophobic character. According to PCA, antioxidant and antidiabetic activity are associated with the content and the in vitro bioaccessibility of most individual and total polyphenols. We can conclude that the in vitro bioaccessibility of the ginkgo phytochemicals from standardized extract tablets depends on whether they are administered alone or combined with casein, glucose, or olive oil. Our results are a good base for future in vivo investigation of bioavailability on animals and humans and they suggest that a different approach should be taken when combining ginkgo tablets and food depending on what biological effect we want to achieve.

## Figures and Tables

**Table 1:** In vitro bioaccessibility (%) of polyphenols (total phenols (TP), flavonoids (TF), phenolic acids (TPA), hydroxycinnamic acids (THA), flavonols (TFLO), flavanols (TFLA), proanthocyanidins (TPAN), quercetin (Q), kaempferol (K), isoramnetin (IzoR), and total identified flavones (TiF)) and triterpene lactones (triterpene lactones expressed in gingkolide A (TTL-A) and B (TTL-B) equivalents).

**In Vitro Bioaccessibility (%) Salivary Phase**	**TP**	**TF**	**TPA**	**THA**	**TFLO**	**TFLA**	**TPAN**	**TTL (GIN-A)**	**TTL (GIN-B)**	**Q**	**K**	**IzoR**	**TiF**
GB + water	178.54 ± 0.37 ^a^	98.46 ± 2.73 ^b^	65.85 ± 13.60 ^bc^	80.08 ± 14.69 ^ab^	79.50 ± 13.87 ^b^	108.11 ± 7.48 ^b^	105.64 ± 15.77 ^b^	99.33 ± 5.22 ^ab^	98.21 ± 14.00 ^ab^	68.45 ± 1.27 ^d^	70.48 ± 2.17 ^c^	82.23 ± 1.34 ^c^	70.38 ± 1.70 ^c^
GB + 40 mg/mL casein	106.20 ± 1.09 ^c^	107.28 ± 3.41 ^a^	131.89 ± 20.68 ^a^	76.47 ± 2.05 ^b^	81.10 ± 4.71 ^b^	70.26 ± 2.13 ^c^	57.93 ± 3.42 ^c^	101.01 ± 0.49 ^ab^	102.41 ± 1.17 ^ab^	97.92 ± 4.01 ^c^	100.09 ± 4.43 ^b^	100.41 ± 1.08 ^b^	102.08 ± 0.13 ^b^
GB + 25 mg/mL glucose	112.52 ± 4.98 ^c^	90.04 ± 2.34 ^b^	40.44 ± 3.00 ^c^	105.60 ± 2.26 ^a^	95.96 ± 6.81 ^ab^	77.75 ± 0.48 ^c^	68.62 ± 8.10 ^bc^	90.17 ± 4.33 ^b^	75.14 ± 10.95 ^b^	113.13 ± 0.46 ^a^	108.76 ± 1.56 ^ab^	103.58 ± 0.10 ^a^	110.26 ± 0.96 ^a^
GB + 5% olive oil	136.74 ± 0.53 ^b^	107.67 ± 5.24 ^a^	100.73 ± 5.26 ^ab^	86.55 ± 10.06 ^ab^	107.72 ± 11.25 ^a^	142.46 ± 1.74 ^a^	162.19 ± 21.15 ^a^	102.53 ± 4.51 ^a^	105.73 ± 10.20 ^a^	104.17 ± 0.06 ^b^	111.11 ± 4.67 ^a^	103.68 ± 0.92 ^a^	105.66 ± 2.04 ^b^
**In Vitro Bioaccessibility (%) Gastric Phase**	**TP**	**TF**	**TPA**	**THA**	**TFLO**	**TFLA**	**TPAN**	**TTL (GIN-A)**	**TTL (GIN-B)**	**Q**	**K**	**IzoR**	**TiF**
GB + water	168.47 ± 6.76 ^a^	117.73 ± 5.89 ^a^	84.53 ± 1.98 ^b^	75.19 ± 2.35 ^ab^	77.78 ± 1.28 ^b^	48.65 ± 6.15 ^c^	35.05 ± 3.29 ^c^	104.49 ± 1.09 ^a^	112.02 ± 2.93 ^a^	62.60 ± 0.28 ^c^	64.08 ± 0.15 ^b^	77.93 ± 0.35 ^b^	64.38 ± 0.18 ^d^
GB + 40 mg/mL casein	156.03 ± 6.92 ^a^	99.73 ± 2.42 ^b^	122.56 ± 23.90 ^a^	67.26 ± 8.47 ^b^	70.01 ± 7.87 ^b^	75.83 ± 0.15 ^b^	51.55 ± 8.21 ^bc^	92.85 ± 0.71 ^b^	82.92 ± 1.70 ^b^	79.55 ± 1.95 ^b^	84.26 ± 0.54 ^a^	92.75 ± 0.45 ^a^	85.45 ± 0.90 ^c^
GB + 25 mg/mL glucose	49.05 ± 0.22 ^b^	105.06 ± 2.03 ^b^	106.99 ± 4.52 ^ab^	81.80 ± 2.22 ^a^	79.04 ± 2.46 ^b^	67.30 ± 5.72 ^b^	77.86 ± 10.13 ^b^	89.48 ± 1.21 ^b^	73.42 ± 3.06 ^c^	98.76 ± 3.29 ^a^	86.51 ± 4.73 ^a^	95.67 ± 2.42 ^a^	92.61 ± 0.65 ^a^
GB + 5% olive oil	41.46 ± 3.40 ^b^	125.53 ± 5.00 ^a^	112.37 ± 6.48 ^ab^	73.96 ± 0.59 ^ab^	95.07 ± 0.58 ^a^	100.77 ± 4.57 ^a^	118.84 ± 19.86 ^a^	91.24 ± 1.95 ^b^	80.18 ± 4.40 ^bc^	94.11 ± 0.25 ^a^	88.66 ± 1.76 ^a^	91.80 ± 2.40 ^a^	89.83 ± 0.80 ^b^
**In Vitro Bioaccessibility (%) Intestinal Phase**	**TP**	**TF**	**TPA**	**THA**	**TFLO**	**TFLA**	**TPAN**	**TTL (GIN-A)**	**TTL (GIN-B)**	**Q**	**K**	**IzoR**	**TiF**
GB + water	83.81 ± 6.85 ^a^	106.24 ± 3.77 ^ab^	51.96 ± 12.02 ^c^	86.93 ± 1.82 ^a^	84.46 ± 1.85 ^b^	45.48 ± 6.98 ^c^	33.33 ± 3.81 ^c^	92.32 ± 5.18 ^bc^	79.41 ± 13.89 ^bc^	63.49 ± 0.33 ^c^	69.47 ± 3.72 ^c^	84.74 ± 0.15 ^b^	67.80 ± 1.59 ^c^
GB + 40 mg/mL casein	64.07 ± 1.43 ^b^	112.24 ± 0.78 ^a^	167.23 ± 4.16 ^a^	72.98 ± 4.81 ^b^	70.89 ± 4.96 ^c^	69.30 ± 1.15 ^ab^	76.85 ± 3.48 ^b^	96.08 ± 1.05 ^ab^	90.64 ± 2.51 ^ab^	77.29 ± 4.01 ^b^	83.32 ± 5.66 ^b^	92.13 ± 4.09 ^a^	83.74 ± 4.92 ^b^
GB + 25 mg/mL glucose	61.83 ± 1.05 ^b^	92.61 ± 4.05 ^c^	61.72 ± 10.15 ^c^	85.03 ± 0.63 ^a^	79.62 ± 0.91 ^b^	59.44 ± 10.65 ^bc^	32.81 ± 4.60 ^c^	86.77 ± 0.64 ^c^	66.56 ± 1.62 ^c^	96.50 ± 1.18 ^a^	96.47 ± 0.44 ^a^	95.10 ± 0.51 ^a^	96.31 ± 0.84 ^a^
GB + 5% olive oil	55.17 ± 10.70 ^b^	96.60 ± 4.65 ^bc^	103.97 ± 11.37 ^b^	76.10 ± 3.79 ^b^	95.33 ± 3.91 ^a^	83.33 ± 2.39 ^a^	100.67 ± 0.17 ^a^	98.74 ± 0.76 ^a^	97.15 ± 1.71 ^a^	92.77 ± 3.10 ^a^	89.29 ± 0.49 ^ab^	95.01 ± 1.16 ^a^	89.89±1.25 ^ab^

Values represent mean ± SD of 3 replicates. Different letters indicate significant difference at *p* ≤ 0.05. The statistics were carried out separately for digested samples at each stage of digestion (separately for salivary phase, gastric phase, and intestinal phase).

## Data Availability

The original contributions presented in this study are included in the article/Supplementary Material. Further in-quiries can be directed to the corresponding author.

## Supplementary Materials

The following supporting information can be downloaded at: https://www.mdpi.com/article/10.3390/molecules29225300/s1, Figure S1: HPLC chromatograms, recorded at 360 nm, of (A) ginkgo extract, and (B) solution containing standards quercetin, kaempferol and isorhamnetin. Q = quercetin, K = kaempferol, and Isorh = isorhamnetin.
