# Supplementary figures and images for: Effect of Protein, Carbohydrate, and Oil on Phytochemical Bioaccessibility and Bioactivities of the Ginkgo biloba L. Leaf Formulations After In Vitro Digestion
Source: Molecules. 2024 Nov 9;29(22):5300. doi: 10.3390/molecules29225300 (PMC11596269; doi:10.3390/molecules29225300)

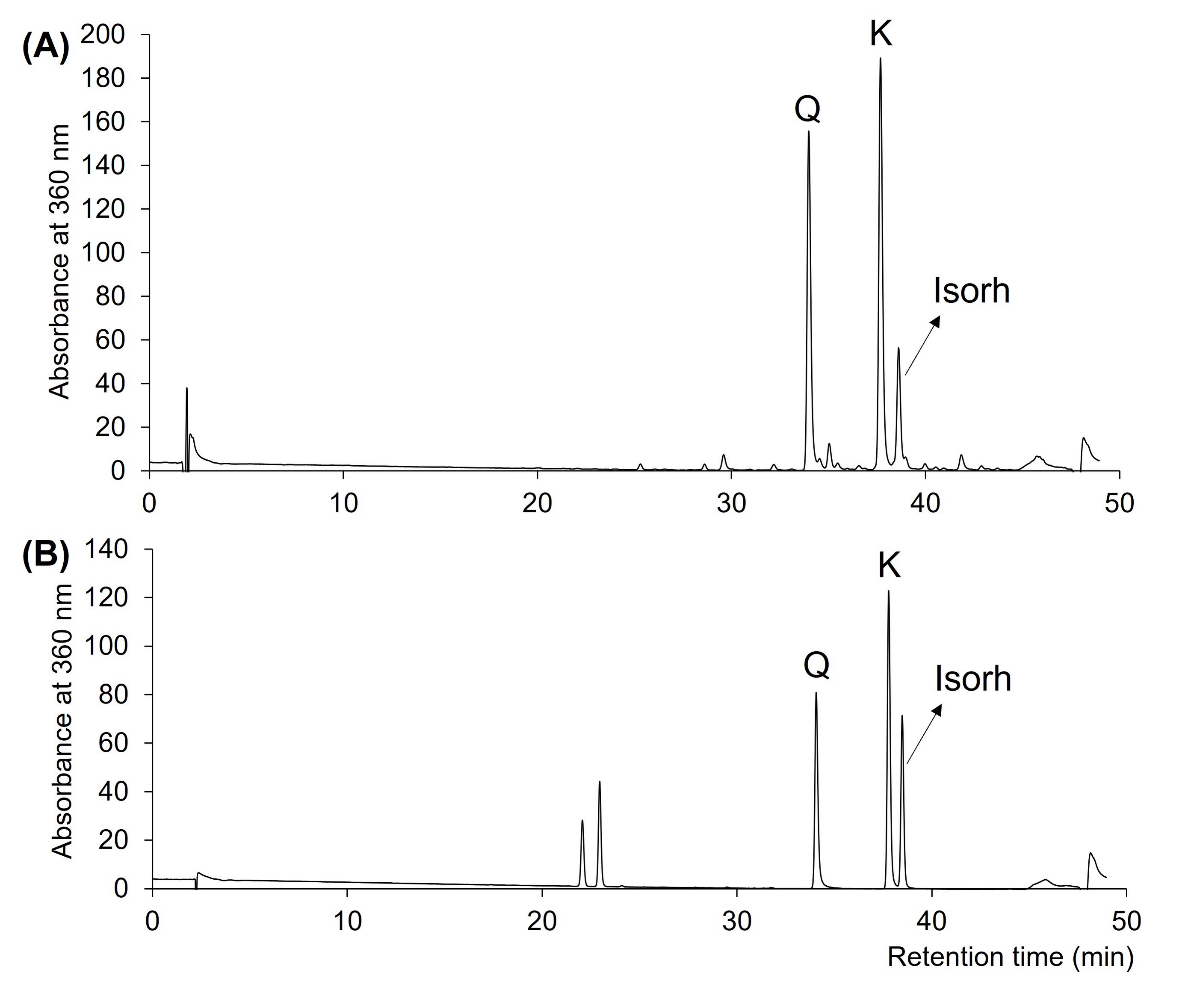

Supplement: Supplementary file 1 [file molecules-29-05300-s001.zip › molecules-3293316-supplementary.jpg]
